# (*E*)-1-(3,4-Di­meth­oxy­phen­yl)-3-(1,3-diphenyl-1*H*-pyrazol-4-yl)prop-2-en-1-one

**DOI:** 10.1107/S2414314624008666

**Published:** 2024-09-06

**Authors:** Jiha Sung

**Affiliations:** a Department of Applied Chemistry Dongduk Women’s University Seoul 136-714 Republic of Korea; University of Aberdeen, United Kingdom

**Keywords:** crystal structure, pyrazole, chalcone, C—H⋯O hydrogen bonds

## Abstract

In the title compound, C_26_H_22_N_2_O_3_, the dihedral angle between the benzene and pyrazole rings of the chalcone unit is 88.3 (1)°. The pyrazole ring has two attached phenyl rings that form dihedral angles with the pyrazole ring of 22.6 (2) and 40.0 (1)°. In the crystal, pairwise C—H⋯O hydrogen bonds generate *R*^2^_2_(20) inversion dimers.

## Abstract 2

The crystal structure of a pyrazole chalcone is reported.

## Graphical abstract

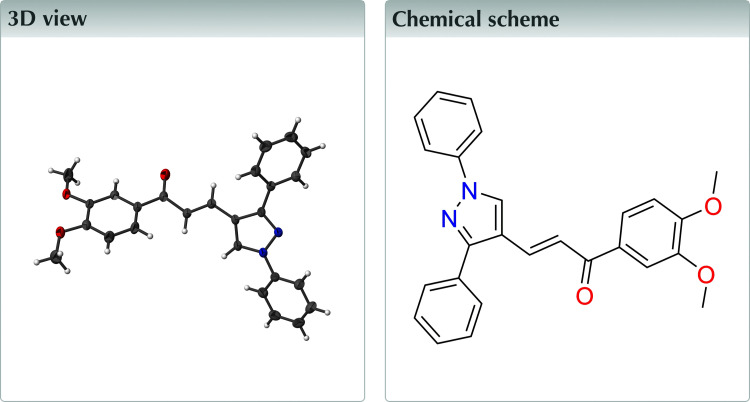

## Structure description

Pyrazoles are promising scaffolds in medicinal chemistry due to their versatile biological efficacy, including anti­bacterial, anti-inflammatory, anti­oxidant, anti­depressant, and anti­cancer activity (Brullo *et al.*, 2020 ▸; Ebenezer *et al.*, 2022 ▸). As a result of intensive research on the anti­cancer activity of pyrazole-containing mol­ecules, a number of anti­cancer drugs such as niraparib, critinib and darolutamide have been developed and are commercially available (Sivaramakarthikeyan *et al.*, 2020 ▸). Chalcones also exhibit various physiological phenomena, including anti­cancer activity (Elkanzi *et al.*, 2022 ▸). As part of our ongoing research to develop new chalcones derivatives (Sung, 2019 ▸) with anti-cancer activities, the pyrazole-containing chalcone title compound was synthesized and its crystal structure was determined.

**Figure 1:**
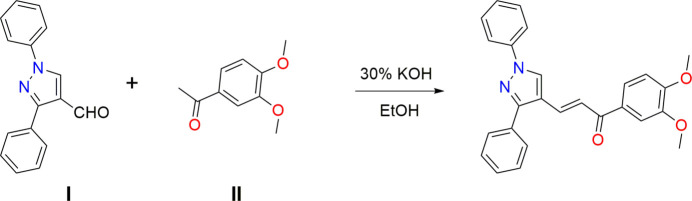
Synthetic scheme for the title compound.

**Figure 2:**
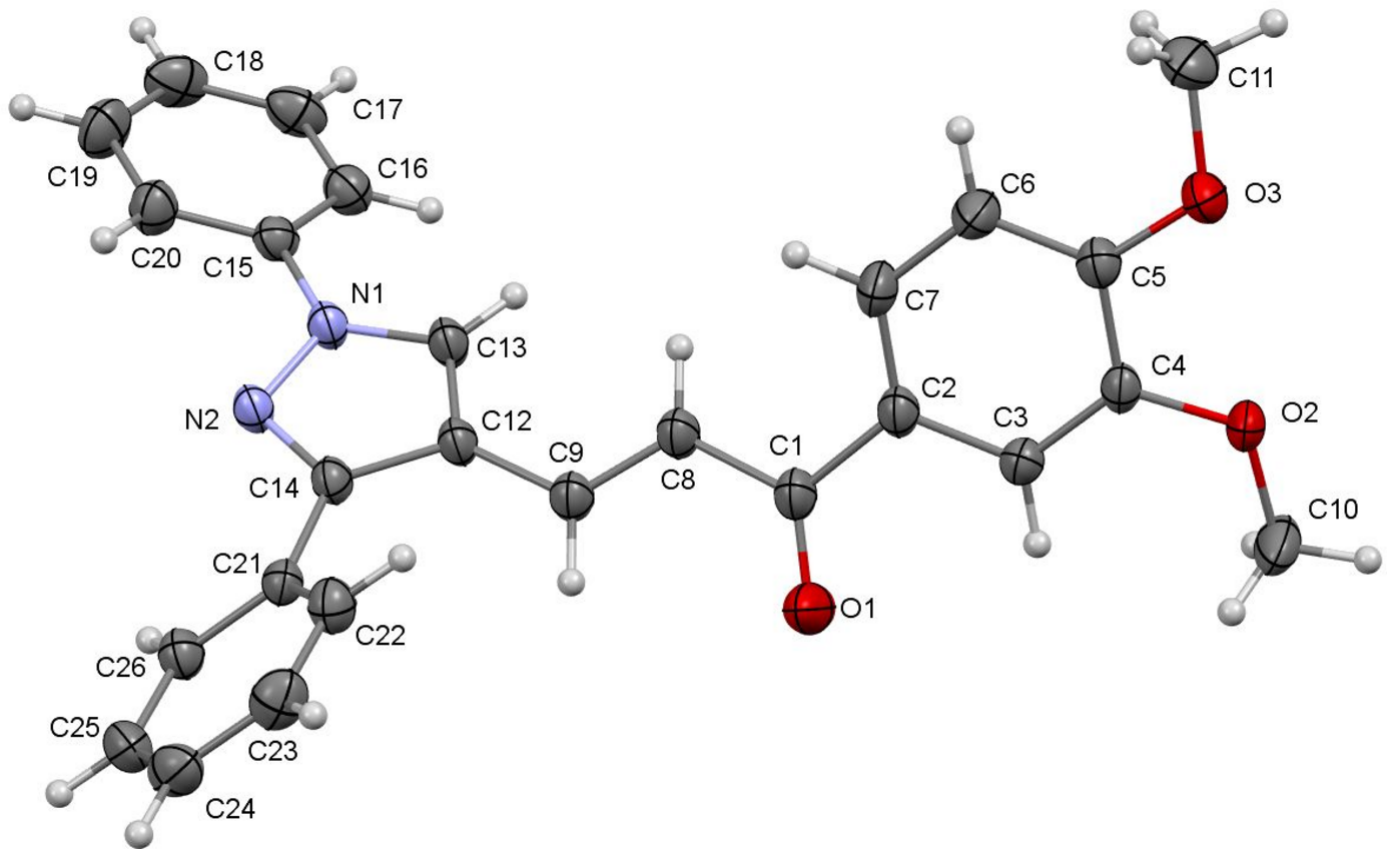
The mol­ecular structure of the title compound, showing displacement ellipsoids drawn at the 50% probability level.

**Figure 3:**
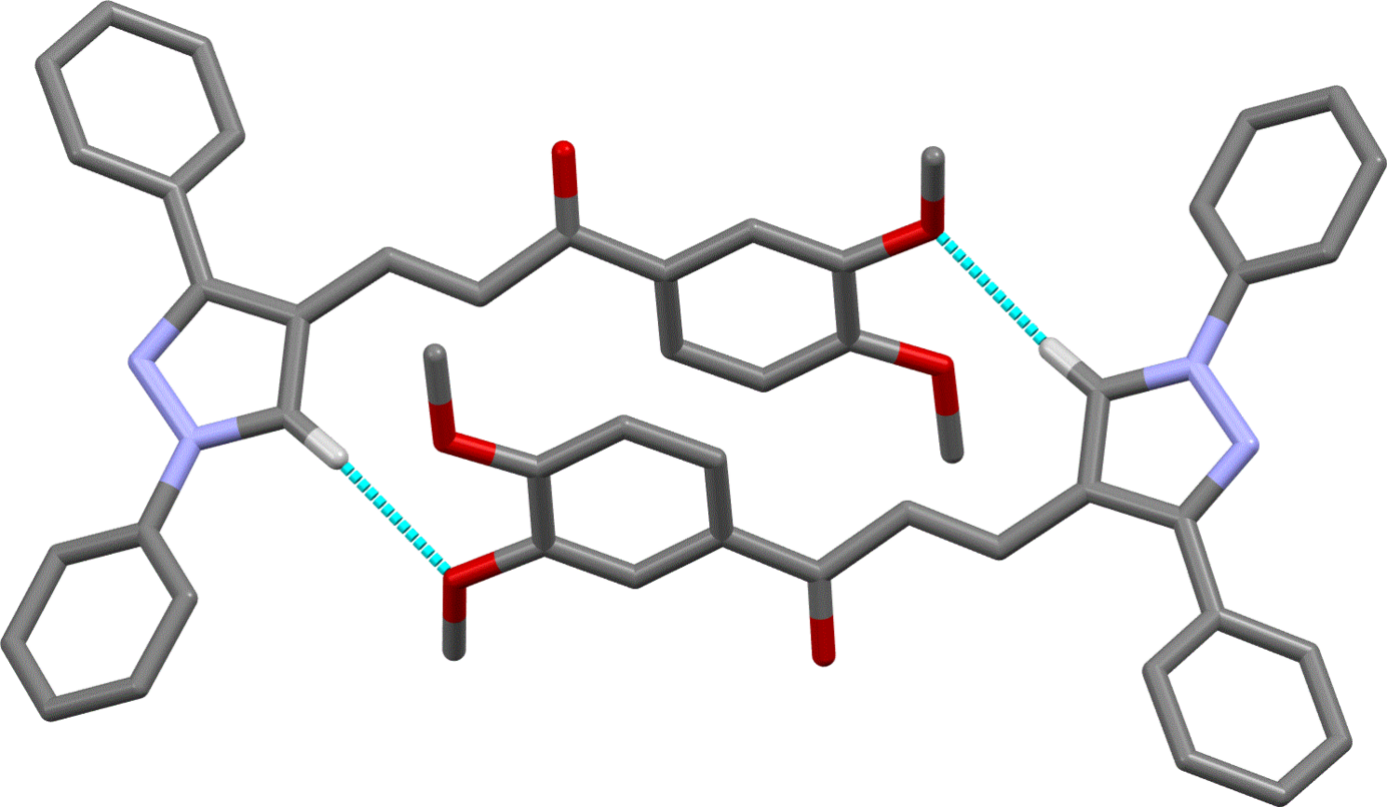
Part of the crystal structure of the title compound, showing the weak C—H⋯O pairwise hydrogen bonds that form 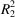
(20) dimers.

The title compound, C_26_H_22_N_2_O_3_, was prepared by a Claisen–Schmidt condensation reaction between 3,4-dimehoxyaceo­phenone and 1,3-diphenyl-1*H*-pyrazole-4-carbaldehyde (Fig. 1 ▸). The mol­ecular structure of title compound is shown in Fig. 2 ▸. The C=C and C=O double bonds usually lie in the same plane in the enone bridge of the chalcone unit. However, in this mol­ecule, the O1—C1—C8—C9 torsion angle is 17.3 (2)°, indicating a significant twist. The C2–C7 benzene ring has two meth­oxy groups attached at C4 and C5, which are twisted from the ring plane with torsion angles of 6.1 (2)° [C3—C4—O2—C10] and −7.3 (2)° [C6—C5—O3—C11]. The pyrazole ring (N1/N2/C14/C12/C13) has the C15–C20 and C21–C26 phenyl groups attached to atoms N1 and C14, respectively. The C15–C20 and C21–C26 phenyl rings make dihedral angles with the pyrazole ring of 22.6 (2) and 40.0 (1)°, respectively, while the dihedral angle between the phenyl rings is 53.3 (3)°. In the crystal, pairs of C—H—O hydrogen bonds generate inversion dimers with graph-set notation 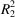 (20) (Table 1 ▸, Fig. 3 ▸).

## Synthesis and crystallization

3,4-Di­meth­oxy­aceo­phenone (180 mg, 1 mmol) was dissolved in 20 ml of ethanol. Then, 1,3-diphenyl-1*H*-pyrazole-4-carbaldehyde (248 mg, 1 mmol) was slowly added until a clear solution was formed. The temperature of reaction mixture was adjusted to 276–277 K using an ice bath. To the cooled reaction mixture was added 1.5 ml of 30% aqueous KOH solution, and the reaction mixture was stirred at room temperature for 30 h. This mixture was poured into iced water (50 ml) and was acidified (pH = 3) with 3 *N* HCl solution to give a precipitate. After filtration, the crude solid was recrystallized from ethanol solution to form crystals in the form of yellow blocks suitable for X-ray diffraction.

## Refinement

Crystal data, data collection and structure refinement details are summarized in Table 2 ▸.

## Supplementary Material

Crystal structure: contains datablock(s) I. DOI: 10.1107/S2414314624008666/hb4483sup1.cif

Structure factors: contains datablock(s) I. DOI: 10.1107/S2414314624008666/hb4483Isup2.hkl

Supporting information file. DOI: 10.1107/S2414314624008666/hb4483Isup3.cml

CCDC reference: 2381555

Additional supporting information:  crystallographic information; 3D view; checkCIF report

## Figures and Tables

**Table 1:** Hydrogen-bond geometry (Å, °)

*D*—H⋯*A*	*D*—H	H⋯*A*	*D*⋯*A*	*D*—H⋯*A*
C13—H13⋯O2^i^	0.94	2.34	3.2648 (19)	169

Symmetry code: (i) 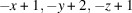.

**Table 2:** Experimental details

Crystal data	
Chemical formula	C_26_H_22_N_2_O_3_
*M*_r_	410.45
Crystal system, space group	Triclinic, *P* 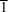
Temperature (K)	223
*a*, *b*, *c* (Å)	9.342 (3), 10.524 (3), 11.967 (4)
α, β, γ (°)	73.831 (10), 79.643 (11), 72.648 (10)
*V* (Å^3^)	1072.6 (6)
*Z*	2
Radiation type	Mo *K*α
μ (mm^−1^)	0.08
Crystal size (mm)	0.26 × 0.22 × 0.07

Data collection	
Diffractometer	PHOTON III M14
Absorption correction	Multi-scan (*SADABS*; Krause *et al.*, 2015 ▸)
*T*_min_, *T*_max_	0.706, 0.746
No. of measured, independent and observed [*I* > 2σ(*I*)] reflections	39655, 5269, 4173
*R*_int_	0.044
(sin θ/λ)_max_ (Å^−1^)	0.667

Refinement	
*R*[*F*^2^ > 2σ(*F*^2^)], *wR*(*F*^2^), *S*	0.044, 0.115, 1.04
No. of reflections	5269
No. of parameters	282
H-atom treatment	H-atom parameters constrained
Δρ_max_, Δρ_min_ (e Å^−3^)	0.24, −0.20

Computer programs: *APEX2* and *SAINT* (Bruker, 2012 ▸), *SHELXS* (Sheldrick, 2008 ▸), *SHELXL2014/7* (Sheldrick, 2015 ▸), *SHELXTL* (Sheldrick, 2008 ▸) and *publCIF* (Westrip, 2010 ▸).

## full crystallographic data

### (E)-1-(3,4-Dimethoxyphenyl)-3-(1,3-diphenyl-1H-pyrazol-4-yl)prop-2-en-1-one Crystal data

**Table:**

C_26_H_22_N_2_O_3_	*Z* = 2
*M**_r_* = 410.45	*F*(000) = 432
Triclinic, *P*1	*D*_x_ = 1.271 Mg m^−^^3^
*a* = 9.342 (3) Å	Mo *K*α radiation, λ = 0.71073 Å
*b* = 10.524 (3) Å	Cell parameters from 9940 reflections
*c* = 11.967 (4) Å	θ = 2.3–28.2°
α = 73.831 (10)°	µ = 0.08 mm^−^^1^
β = 79.643 (11)°	*T* = 223 K
γ = 72.648 (10)°	BLOCK, yellow
*V* = 1072.6 (6) Å^3^	0.26 × 0.22 × 0.07 mm

### (E)-1-(3,4-Dimethoxyphenyl)-3-(1,3-diphenyl-1H-pyrazol-4-yl)prop-2-en-1-one Data collection

**Table:**

PHOTON III M14 diffractometer	4173 reflections with *I* > 2σ(*I*)
φ and ω scans	*R*_int_ = 0.044
Absorption correction: multi-scan (SADABS; Krause *et al.*, 2015)	θ_max_ = 28.3°, θ_min_ = 2.1°
*T*_min_ = 0.706, *T*_max_ = 0.746	*h* = −12→12
39655 measured reflections	*k* = −13→13
5269 independent reflections	*l* = −15→15

### (E)-1-(3,4-Dimethoxyphenyl)-3-(1,3-diphenyl-1H-pyrazol-4-yl)prop-2-en-1-one Refinement

**Table:**

Refinement on *F*^2^	Primary atom site location: structure-invariant direct methods
Least-squares matrix: full	Hydrogen site location: inferred from neighbouring sites
*R*[*F*^2^ > 2σ(*F*^2^)] = 0.044	H-atom parameters constrained
*wR*(*F*^2^) = 0.115	*w* = 1/[σ^2^(*F*_o_^2^) + (0.0449*P*)^2^ + 0.3885*P*] where *P* = (*F*_o_^2^ + 2*F*_c_^2^)/3
*S* = 1.04	(Δ/σ)_max_ < 0.001
5269 reflections	Δρ_max_ = 0.24 e Å^−^^3^
282 parameters	Δρ_min_ = −0.20 e Å^−^^3^
0 restraints	

### (E)-1-(3,4-Dimethoxyphenyl)-3-(1,3-diphenyl-1H-pyrazol-4-yl)prop-2-en-1-one Special details

**Table:**

Geometry. All esds (except the esd in the dihedral angle between two l.s. planes) are estimated using the full covariance matrix. The cell esds are taken into account individually in the estimation of esds in distances, angles and torsion angles; correlations between esds in cell parameters are only used when they are defined by crystal symmetry. An approximate (isotropic) treatment of cell esds is used for estimating esds involving l.s. planes.

### (E)-1-(3,4-Dimethoxyphenyl)-3-(1,3-diphenyl-1H-pyrazol-4-yl)prop-2-en-1-one Fractional atomic coordinates and isotropic or equivalent isotropic displacement parameters (Å^2^)

**Table:**

	*x*	*y*	*z*	*U*_iso_*/*U*_eq_	
C1	0.62809 (17)	0.71283 (14)	0.49957 (12)	0.0323 (3)	
C2	0.67643 (16)	0.84296 (13)	0.47121 (12)	0.0289 (3)	
C3	0.70448 (15)	0.90841 (13)	0.35225 (11)	0.0278 (3)	
H3	0.6908	0.8711	0.2934	0.033*	
C4	0.75205 (14)	1.02730 (13)	0.32222 (11)	0.0256 (3)	
C5	0.77516 (16)	1.08293 (13)	0.41023 (12)	0.0289 (3)	
C6	0.74834 (19)	1.01806 (16)	0.52704 (13)	0.0380 (3)	
H6	0.7640	1.0540	0.5861	0.046*	
C7	0.69805 (18)	0.89929 (15)	0.55667 (12)	0.0367 (3)	
H7	0.6785	0.8568	0.6360	0.044*	
C8	0.55681 (16)	0.66700 (14)	0.61955 (12)	0.0305 (3)	
H8	0.5185	0.7306	0.6663	0.037*	
C9	0.54487 (16)	0.53814 (14)	0.66390 (12)	0.0299 (3)	
H9	0.5866	0.4748	0.6172	0.036*	
C10	0.7475 (2)	1.05899 (18)	0.11652 (13)	0.0506 (5)	
H10A	0.8065	0.9658	0.1172	0.076*	
H10B	0.7719	1.1196	0.0427	0.076*	
H10C	0.6410	1.0632	0.1260	0.076*	
C11	0.8254 (2)	1.27120 (18)	0.45672 (15)	0.0502 (4)	
H11A	0.7316	1.2800	0.5078	0.075*	
H11B	0.8386	1.3616	0.4178	0.075*	
H11C	0.9089	1.2194	0.5026	0.075*	
C12	0.47133 (15)	0.49005 (13)	0.77989 (12)	0.0276 (3)	
C13	0.35511 (16)	0.56706 (13)	0.84202 (12)	0.0301 (3)	
H13	0.3103	0.6616	0.8181	0.036*	
C14	0.49721 (15)	0.35452 (13)	0.85328 (11)	0.0254 (3)	
C15	0.19811 (15)	0.51457 (13)	1.03298 (12)	0.0274 (3)	
C16	0.07757 (16)	0.62931 (14)	1.00652 (13)	0.0336 (3)	
H16	0.0756	0.6874	0.9309	0.040*	
C17	−0.04030 (17)	0.65744 (16)	1.09308 (15)	0.0397 (4)	
H17	−0.1219	0.7351	1.0755	0.048*	
C18	−0.03903 (19)	0.57258 (17)	1.20473 (15)	0.0439 (4)	
H18	−0.1196	0.5915	1.2625	0.053*	
C19	0.08327 (19)	0.45892 (18)	1.23016 (14)	0.0440 (4)	
H19	0.0855	0.4014	1.3060	0.053*	
C20	0.20220 (17)	0.42922 (15)	1.14506 (13)	0.0356 (3)	
H20	0.2845	0.3523	1.1631	0.043*	
C21	0.61238 (15)	0.22895 (13)	0.83440 (11)	0.0257 (3)	
C22	0.75946 (16)	0.23372 (14)	0.78641 (12)	0.0324 (3)	
H22	0.7839	0.3184	0.7613	0.039*	
C23	0.86940 (17)	0.11392 (16)	0.77567 (14)	0.0389 (3)	
H23	0.9679	0.1181	0.7436	0.047*	
C24	0.83513 (18)	−0.01186 (15)	0.81191 (13)	0.0395 (4)	
H24	0.9101	−0.0925	0.8046	0.047*	
C25	0.68948 (18)	−0.01789 (14)	0.85903 (13)	0.0371 (3)	
H25	0.6657	−0.1028	0.8837	0.045*	
C26	0.57853 (16)	0.10188 (13)	0.86983 (12)	0.0310 (3)	
H26	0.4800	0.0972	0.9012	0.037*	
N1	0.31718 (13)	0.48180 (11)	0.94375 (10)	0.0276 (2)	
N2	0.40423 (12)	0.34957 (11)	0.95287 (10)	0.0271 (2)	
O1	0.64801 (16)	0.64685 (12)	0.42551 (10)	0.0540 (3)	
O2	0.78144 (12)	1.10062 (9)	0.21009 (8)	0.0311 (2)	
O3	0.82098 (12)	1.20091 (10)	0.37080 (9)	0.0362 (2)	

### (E)-1-(3,4-Dimethoxyphenyl)-3-(1,3-diphenyl-1H-pyrazol-4-yl)prop-2-en-1-one Atomic displacement parameters (Å^2^)

**Table:**

	*U*^11^	*U*^22^	*U*^33^	*U*^12^	*U*^13^	*U*^23^
C1	0.0405 (8)	0.0263 (7)	0.0269 (7)	−0.0117 (6)	0.0029 (6)	−0.0019 (5)
C2	0.0330 (7)	0.0244 (6)	0.0253 (6)	−0.0089 (5)	0.0017 (5)	−0.0012 (5)
C3	0.0329 (7)	0.0256 (6)	0.0234 (6)	−0.0089 (5)	0.0005 (5)	−0.0047 (5)
C4	0.0267 (6)	0.0222 (6)	0.0222 (6)	−0.0045 (5)	0.0012 (5)	−0.0006 (5)
C5	0.0325 (7)	0.0257 (6)	0.0277 (7)	−0.0102 (5)	−0.0007 (5)	−0.0037 (5)
C6	0.0557 (10)	0.0390 (8)	0.0243 (7)	−0.0222 (7)	−0.0021 (6)	−0.0063 (6)
C7	0.0525 (9)	0.0354 (8)	0.0216 (6)	−0.0198 (7)	0.0000 (6)	0.0008 (6)
C8	0.0355 (7)	0.0252 (6)	0.0269 (7)	−0.0091 (5)	0.0039 (6)	−0.0033 (5)
C9	0.0336 (7)	0.0251 (6)	0.0269 (7)	−0.0071 (5)	0.0020 (5)	−0.0035 (5)
C10	0.0866 (14)	0.0502 (10)	0.0237 (7)	−0.0381 (10)	−0.0088 (8)	0.0007 (7)
C11	0.0754 (13)	0.0474 (10)	0.0415 (9)	−0.0368 (9)	0.0012 (8)	−0.0146 (8)
C12	0.0328 (7)	0.0203 (6)	0.0266 (6)	−0.0085 (5)	0.0000 (5)	−0.0014 (5)
C13	0.0361 (7)	0.0208 (6)	0.0289 (7)	−0.0081 (5)	0.0008 (6)	−0.0009 (5)
C14	0.0275 (6)	0.0222 (6)	0.0253 (6)	−0.0090 (5)	−0.0008 (5)	−0.0020 (5)
C15	0.0290 (7)	0.0255 (6)	0.0285 (7)	−0.0102 (5)	0.0020 (5)	−0.0076 (5)
C16	0.0343 (8)	0.0278 (7)	0.0371 (8)	−0.0071 (6)	−0.0020 (6)	−0.0073 (6)
C17	0.0315 (8)	0.0339 (8)	0.0538 (10)	−0.0054 (6)	0.0012 (7)	−0.0180 (7)
C18	0.0381 (8)	0.0480 (9)	0.0481 (9)	−0.0162 (7)	0.0150 (7)	−0.0226 (8)
C19	0.0468 (9)	0.0492 (9)	0.0315 (8)	−0.0161 (8)	0.0083 (7)	−0.0064 (7)
C20	0.0360 (8)	0.0334 (7)	0.0314 (7)	−0.0068 (6)	0.0016 (6)	−0.0040 (6)
C21	0.0298 (7)	0.0237 (6)	0.0208 (6)	−0.0063 (5)	−0.0023 (5)	−0.0020 (5)
C22	0.0322 (7)	0.0310 (7)	0.0313 (7)	−0.0098 (6)	−0.0012 (6)	−0.0030 (6)
C23	0.0297 (7)	0.0439 (9)	0.0357 (8)	−0.0026 (6)	−0.0002 (6)	−0.0075 (7)
C24	0.0425 (9)	0.0327 (8)	0.0347 (8)	0.0061 (6)	−0.0071 (7)	−0.0096 (6)
C25	0.0512 (9)	0.0237 (7)	0.0338 (8)	−0.0071 (6)	−0.0055 (7)	−0.0050 (6)
C26	0.0344 (7)	0.0268 (7)	0.0304 (7)	−0.0100 (6)	−0.0013 (6)	−0.0039 (5)
N1	0.0308 (6)	0.0202 (5)	0.0271 (6)	−0.0055 (4)	0.0004 (5)	−0.0019 (4)
N2	0.0288 (6)	0.0197 (5)	0.0279 (6)	−0.0051 (4)	0.0008 (4)	−0.0020 (4)
O1	0.0929 (10)	0.0427 (6)	0.0342 (6)	−0.0384 (7)	0.0159 (6)	−0.0134 (5)
O2	0.0460 (6)	0.0264 (5)	0.0203 (4)	−0.0153 (4)	−0.0013 (4)	0.0004 (4)
O3	0.0517 (6)	0.0316 (5)	0.0299 (5)	−0.0223 (5)	0.0001 (5)	−0.0050 (4)

### (E)-1-(3,4-Dimethoxyphenyl)-3-(1,3-diphenyl-1H-pyrazol-4-yl)prop-2-en-1-one Geometric parameters (Å, º)

**Table:**

C1—O1	1.2283 (18)	C13—N1	1.3560 (17)
C1—C8	1.4832 (19)	C13—H13	0.9400
C1—C2	1.4998 (19)	C14—N2	1.3408 (17)
C2—C7	1.386 (2)	C14—C21	1.4784 (18)
C2—C3	1.4133 (18)	C15—C16	1.390 (2)
C3—C4	1.3842 (18)	C15—C20	1.391 (2)
C3—H3	0.9400	C15—N1	1.4302 (17)
C4—O2	1.3743 (15)	C16—C17	1.393 (2)
C4—C5	1.4139 (19)	C16—H16	0.9400
C5—O3	1.3659 (16)	C17—C18	1.385 (2)
C5—C6	1.3884 (19)	C17—H17	0.9400
C6—C7	1.397 (2)	C18—C19	1.391 (2)
C6—H6	0.9400	C18—H18	0.9400
C7—H7	0.9400	C19—C20	1.390 (2)
C8—C9	1.3422 (18)	C19—H19	0.9400
C8—H8	0.9400	C20—H20	0.9400
C9—C12	1.4588 (19)	C21—C26	1.3988 (18)
C9—H9	0.9400	C21—C22	1.4005 (19)
C10—O2	1.4249 (19)	C22—C23	1.390 (2)
C10—H10A	0.9700	C22—H22	0.9400
C10—H10B	0.9700	C23—C24	1.388 (2)
C10—H10C	0.9700	C23—H23	0.9400
C11—O3	1.4363 (19)	C24—C25	1.389 (2)
C11—H11A	0.9700	C24—H24	0.9400
C11—H11B	0.9700	C25—C26	1.394 (2)
C11—H11C	0.9700	C25—H25	0.9400
C12—C13	1.3817 (19)	C26—H26	0.9400
C12—C14	1.4274 (17)	N1—N2	1.3719 (15)
			
O1—C1—C8	121.61 (13)	N2—C14—C12	111.62 (11)
O1—C1—C2	120.66 (12)	N2—C14—C21	119.59 (11)
C8—C1—C2	117.73 (12)	C12—C14—C21	128.73 (12)
C7—C2—C3	118.98 (12)	C16—C15—C20	120.49 (13)
C7—C2—C1	122.70 (12)	C16—C15—N1	119.88 (12)
C3—C2—C1	118.31 (12)	C20—C15—N1	119.61 (12)
C4—C3—C2	120.18 (12)	C15—C16—C17	119.35 (14)
C4—C3—H3	119.9	C15—C16—H16	120.3
C2—C3—H3	119.9	C17—C16—H16	120.3
O2—C4—C3	125.50 (12)	C18—C17—C16	120.93 (14)
O2—C4—C5	114.22 (11)	C18—C17—H17	119.5
C3—C4—C5	120.28 (12)	C16—C17—H17	119.5
O3—C5—C6	125.18 (13)	C17—C18—C19	119.00 (14)
O3—C5—C4	115.36 (11)	C17—C18—H18	120.5
C6—C5—C4	119.45 (12)	C19—C18—H18	120.5
C5—C6—C7	119.92 (14)	C20—C19—C18	120.95 (15)
C5—C6—H6	120.0	C20—C19—H19	119.5
C7—C6—H6	120.0	C18—C19—H19	119.5
C2—C7—C6	121.18 (13)	C19—C20—C15	119.26 (14)
C2—C7—H7	119.4	C19—C20—H20	120.4
C6—C7—H7	119.4	C15—C20—H20	120.4
C9—C8—C1	122.51 (13)	C26—C21—C22	118.70 (12)
C9—C8—H8	118.7	C26—C21—C14	120.51 (12)
C1—C8—H8	118.7	C22—C21—C14	120.71 (12)
C8—C9—C12	124.25 (13)	C23—C22—C21	120.31 (13)
C8—C9—H9	117.9	C23—C22—H22	119.8
C12—C9—H9	117.9	C21—C22—H22	119.8
O2—C10—H10A	109.5	C24—C23—C22	120.61 (14)
O2—C10—H10B	109.5	C24—C23—H23	119.7
H10A—C10—H10B	109.5	C22—C23—H23	119.7
O2—C10—H10C	109.5	C23—C24—C25	119.64 (13)
H10A—C10—H10C	109.5	C23—C24—H24	120.2
H10B—C10—H10C	109.5	C25—C24—H24	120.2
O3—C11—H11A	109.5	C24—C25—C26	120.07 (14)
O3—C11—H11B	109.5	C24—C25—H25	120.0
H11A—C11—H11B	109.5	C26—C25—H25	120.0
O3—C11—H11C	109.5	C25—C26—C21	120.66 (14)
H11A—C11—H11C	109.5	C25—C26—H26	119.7
H11B—C11—H11C	109.5	C21—C26—H26	119.7
C13—C12—C14	104.13 (11)	C13—N1—N2	111.93 (11)
C13—C12—C9	126.94 (12)	C13—N1—C15	127.64 (11)
C14—C12—C9	128.89 (12)	N2—N1—C15	120.36 (10)
N1—C13—C12	107.76 (12)	C14—N2—N1	104.55 (10)
N1—C13—H13	126.1	C4—O2—C10	117.79 (11)
C12—C13—H13	126.1	C5—O3—C11	117.12 (11)
			
O1—C1—C2—C7	−160.59 (16)	C16—C17—C18—C19	0.8 (2)
C8—C1—C2—C7	19.1 (2)	C17—C18—C19—C20	−0.7 (3)
O1—C1—C2—C3	17.8 (2)	C18—C19—C20—C15	−0.1 (2)
C8—C1—C2—C3	−162.53 (13)	C16—C15—C20—C19	0.8 (2)
C7—C2—C3—C4	−0.3 (2)	N1—C15—C20—C19	−177.60 (14)
C1—C2—C3—C4	−178.78 (12)	N2—C14—C21—C26	39.32 (19)
C2—C3—C4—O2	−179.35 (12)	C12—C14—C21—C26	−143.64 (14)
C2—C3—C4—C5	1.0 (2)	N2—C14—C21—C22	−137.32 (14)
O2—C4—C5—O3	0.76 (17)	C12—C14—C21—C22	39.7 (2)
C3—C4—C5—O3	−179.53 (12)	C26—C21—C22—C23	−0.7 (2)
O2—C4—C5—C6	179.67 (13)	C14—C21—C22—C23	176.01 (13)
C3—C4—C5—C6	−0.6 (2)	C21—C22—C23—C24	0.2 (2)
O3—C5—C6—C7	178.41 (14)	C22—C23—C24—C25	0.2 (2)
C4—C5—C6—C7	−0.4 (2)	C23—C24—C25—C26	0.0 (2)
C3—C2—C7—C6	−0.7 (2)	C24—C25—C26—C21	−0.5 (2)
C1—C2—C7—C6	177.69 (14)	C22—C21—C26—C25	0.8 (2)
C5—C6—C7—C2	1.1 (2)	C14—C21—C26—C25	−175.86 (13)
O1—C1—C8—C9	17.3 (2)	C12—C13—N1—N2	0.22 (16)
C2—C1—C8—C9	−162.38 (14)	C12—C13—N1—C15	−176.70 (13)
C1—C8—C9—C12	−177.91 (13)	C16—C15—N1—C13	21.0 (2)
C8—C9—C12—C13	28.4 (2)	C20—C15—N1—C13	−160.54 (14)
C8—C9—C12—C14	−154.16 (15)	C16—C15—N1—N2	−155.67 (12)
C14—C12—C13—N1	0.05 (15)	C20—C15—N1—N2	22.77 (19)
C9—C12—C13—N1	178.01 (13)	C12—C14—N2—N1	0.43 (15)
C13—C12—C14—N2	−0.31 (16)	C21—C14—N2—N1	177.95 (11)
C9—C12—C14—N2	−178.22 (13)	C13—N1—N2—C14	−0.40 (15)
C13—C12—C14—C21	−177.54 (13)	C15—N1—N2—C14	176.77 (12)
C9—C12—C14—C21	4.5 (2)	C3—C4—O2—C10	6.1 (2)
C20—C15—C16—C17	−0.7 (2)	C5—C4—O2—C10	−174.22 (14)
N1—C15—C16—C17	177.73 (13)	C6—C5—O3—C11	−7.3 (2)
C15—C16—C17—C18	−0.2 (2)	C4—C5—O3—C11	171.50 (14)

### (E)-1-(3,4-Dimethoxyphenyl)-3-(1,3-diphenyl-1H-pyrazol-4-yl)prop-2-en-1-one Hydrogen-bond geometry (Å, º)

**Table:**

*D*—H···*A*	*D*—H	H···*A*	*D*···*A*	*D*—H···*A*
C13—H13···O2^i^	0.94	2.34	3.2648 (19)	169

Symmetry code: (i) −*x*+1, −*y*+2, −*z*+1.
